# Which Is in Front of Chinese People, Past or Future? The Effect of Language and Culture on Temporal Gestures and Spatial Conceptions of Time

**DOI:** 10.1111/cogs.12804

**Published:** 2019-12-11

**Authors:** Yan Gu, Yeqiu Zheng, Marc Swerts

**Affiliations:** ^1^ Tilburg Center for Cognition and Communication Tilburg University; ^2^ Department of Experimental Psychology University College London; ^3^ Department of Econometrics and Operations Research Tilburg University

**Keywords:** Temporal‐focus hypothesis, Gesture and conceptual metaphor, Space and time, Language and thought, Cross‐cultural differences, Chinese

## Abstract

The *temporal‐focus hypothesis* claims that whether people conceptualize the past or the future as in front of them depends on their cultural attitudes toward time; such conceptualizations can be independent from the space–time metaphors expressed through language. In this paper, we study how Chinese people conceptualize time on the sagittal axis to find out the respective influences of language and culture on mental space–time mappings. An examination of Mandarin speakers' co‐speech gestures shows that some Chinese spontaneously perform past‐in‐front/future‐at‐back (besides future‐in‐front/past‐at‐back) gestures, especially when gestures are accompanying past‐in‐front/future‐at‐back space–time metaphors (Exp. 1). Using a temporal performance task, the study confirms that Chinese can conceptualize the future as behind and the past as in front of them, and that such space–time mappings are affected by the different expressions of Mandarin space–time metaphors (Exp. 2). Additionally, a survey on cultural attitudes toward time shows that Chinese tend to focus slightly more on the future than on the past (Exp. 3). Within the Chinese sample, we did not find evidence for the effect of participants' cultural temporal attitudes on space–time mappings, but a cross‐cultural comparison of space–time mappings between Chinese, Moroccans, and Spaniards provides strong support for the *temporal‐focus hypothesis*. Furthermore, the results of Exp. 2 are replicated even after controlling for factors such as cultural temporal attitudes and age (Exp. 3), which implies that linguistic sagittal temporal metaphors can indeed influence Mandarin speakers' space–time mappings. The findings not only contribute to a better understanding of Chinese people's sagittal temporal orientation, but also have additional implications for theories on the mental space–time mappings and the relationship between language and thought.

## 1 Introduction

Across cultures people use space to represent time (Bottini et al., 2015; Casasanto & Boroditsky, 2008; see reviews Bender & Beller, 2014; Núñez & Cooperrider, 2013). The concepts of future and past are often linguistically expressed by the use of spatial metaphors. For instance, in English, we look *forward* to the bright future lying *ahead*, or look *back* to the hard times *behind* (e.g., Clark, 1973; Lakoff & Johnson, 1980). Interestingly, studies have shown that many people not only talk about time using a front‐back axis, but also tend to think about time this way, that is, the past is mentally “behind,” and the future “ahead” of the speaker (Boroditsky, 2000; Miles, Nind, & Macrae, 2010; Ulrich et al., 2012). This particular conceptualization seems to be consistent with the bodily experience of walking in a certain direction, so that the path that we have passed by is the past and the place that we are heading toward is the future (e.g., Clark, 1973).

Despite this general tendency in languages like English, speakers of other languages may exhibit opposite sagittal space–time mappings than the one explained above. For example, Aymara speakers can conceptualize the past as seen events in front of them, and the future as yet unseen events behind them, which is reflected in the observation that their words for *past* and *future* also mean *front/seen* and *back/unseen* (e.g., *front year* means *last year*). This past‐in‐front mapping is also apparent from Aymara's temporal gestures (Núñez & Sweetser, 2006).

Somewhat similar to Aymara is Mandarin Chinese, in which sagittal words for spatial “front” (前/qián)” and “back” (后/hòu) are also used as temporal conceptions of “before/past” and “after/future.” Such sagittal spatial metaphors for time suggest past‐in‐front/future‐at‐back space–time mappings (Example 1, Table 1). However, how Mandarin speakers conceptualize or gesture about time using the front‐back space is barely known (Yu, 2012). Based on the first attempt by Fuhrman et al. (2011), Xiao, Zhao, and Chen (2018)'s recent study aims to provide a comprehensive picture of the psychological reality of time by Mandarin speakers.

**Table 1:** Examples of Mandarin phrases suggesting past‐in‐front/future‐at‐back mappings (1) and future‐in‐front/past‐at‐back mappings (2)

Example (1)	后/qián	天/tiān,	今/jīn	后/hòu
	back	day,	today	back
	the day after tomorrow,		from now on	
Example (2)	展/zhăn	望/wàng	未/wèi	来/lái
	unfold	gaze‐into‐distance	hasn't	come
	Looking far ahead/into the future.			
	回/huí	首/shǒu	过/guò	去/qù
	turn‐around	head	pass	go
	Looking back to the past.			

Xiao et al., (2018) (also Yu, 2012) propose that, like English, there are two kinds of time perspective‐taking for Chinese people, related to a moving‐ego and moving‐time perspective (e.g., Clark, 1973; Gentner, Imai, & Boroditsky, 2002; Moore, 2011; Núñez, Motz, & Teuscher, 2006; Walker, Bergen, & Núñez, 2017). They argue that for the Mandarin temporal expressions such as “前天/qián‐tiān” (“front day,” the day before yesterday) and “后天/hòu‐tiān (“back day,” the day after tomorrow), the reference point is not the observer but time (i.e., today) (earlier‐times in‐front‐of later‐times metaphor). By contrast, temporal expressions such as “过去/guò qù” (pass go, past) and “未/将来/wèi/jiāng lái” (hasn't come yet/will come, future, Example 2, Table 1) take the observer as a reference point, suggesting that future is ahead of and the past behind the ego (Yu, 2012) (front‐to‐the‐future metaphor).

**Figure 1:**
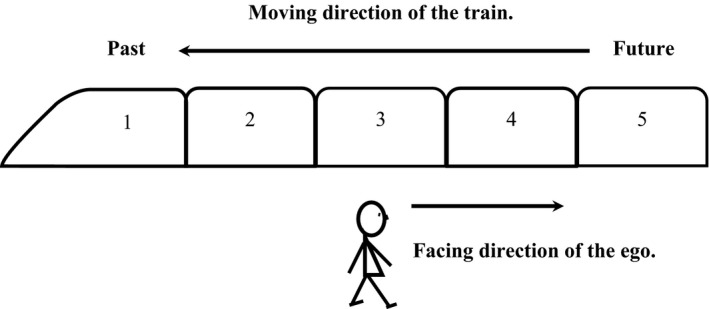
According to Xiao, Zhao, and Chen (2018), time can be perceived as moving from the future to the past, where the ego faces the future (carriage 3 is now): From the time‐reference‐point perspective, earlier events (e.g., carriages 1 and 2) are in front of later events (e.g., carriages 4 and 5), thus the past is in the front of the timeline; from the ego‐reference‐point perspective (*stationary* or *moving*), earlier events are behind the ego, so the past is at the back of the ego.

Xiao et al., (2018) used an illustrative example of a train to explain the sagittal temporal representation of Mandarin speakers based on linguistic analyses (Fig. 1). Time in this visualization would be analogous to a moving train with a number of carriages. The moving‐time perspective (the train) refers to the relation among time points (carriages), whereby earlier time points (e.g., carriages 1 and 2) are in front of later time points (e.g., carriages 4 and 5, from the perspective of the train). The ego‐moving time perspective refers to the relation between the ego (observer) and the time points, with a direction of the future (e.g., carriage 5) in front of the ego.

Studies have shown that Mandarin space–time metaphors not only suggest different temporal perspectives, but may also have an impact on Mandarin speakers' front‐back mental space–time mappings (Fuhrman et al., 2011; Gu, Zheng, & Swerts, 2019; Lai & Boroditsky, 2013). For instance, the Mandarin Chinese lexicon contains both words suggesting past‐at‐back/future‐in‐front and past‐in‐front/future‐at‐back space–time mappings, whereas the sagittal lexical signs of Chinese Sign Language (CSL) do not show this variation as they represent *only* past‐at‐back/future‐in‐front space–time mappings (Wu & Li, 2012; Zheng, 2009). Interestingly, deaf signers of CSL appear to display a different spatio‐temporal reasoning than Mandarin speakers, and Chinese deaf signers with higher written Mandarin proficiency are more likely to have past‐in‐front/future‐at‐back space–time mappings than signers with lower Mandarin proficiency (Gu, Zheng, & Swerts, 2019).

Regardless of any effect of linguistic space–time metaphors on temporal perspectives, Xiao et al., (2018) believe that the ego of a Mandarin speaker *always* faces the future. However, there are also alternative views regarding the metaphorical sagittal orientation of time by Chinese people. For example, some believe that the ego faces the past (Alverson, 1994), whereas others believe that the ego can face both the past and the future (Ahrens & Huang, 2002). Given that an increasing number of studies have shown that humans' mental space–time mappings can be influenced by different factors (e.g., Casasanto & Bottini, 2014; Duffy & Evans, 2017; Duffy, Feist, & McCarthy, 2014; Saj et al., 2014; Santiago et al., 2007; Torralbo, Santiago, & Lupáñez, 2006), it is possible that Chinese people's mental orientation of sagittal time may not only be affected by linguistic space–time metaphors but may also be shaped by additional influences such as culture (e.g., Boroditsky & Gaby, 2010; Floyd, 2016; Fuhrman & Boroditsky, 2010; Le Guen & Balam, 2012; Núñez et al., 2012; Santiago, Román, & Ouellet, 2011).

Indeed, de la Fuente, Santiago, Román, Dumitrache, and Casasanto (2014) propose that people's sagittal mental space–time mappings are not necessarily the exclusive result of the sagittal space–time metaphors expressed in the language, as they can also be influenced more generally by the specific way cultures associate space with time. For instance, there are only future‐in‐front mappings but no deictic past‐in‐front mappings in Spanish language (as confirmed by two native Spanish speakers, one of whom is a linguist). Similarly, there are future‐in‐front mappings but “no such reversed spoken metaphors exist in Arabic” (e.g., Casasanto, 2016, p. 181, also see Hamdi, 2008's corpus‐based study). Interestingly, despite the fact that front‐back time lexical metaphors in Spanish and Arabic both only suggest future‐in‐front mappings, Moroccans have a strong tendency for past‐in‐front mapping, whereas most Spaniards have future‐in‐front mappings. The different space–time mappings between Moroccans and Spaniards have been argued to be related to cross‐cultural differences in temporal focus (*temporal‐focus hypothesis*). It is claimed that people who are past‐focused metaphorically have a tendency to place the past in front of them, “in the location where they could focus on the past literally with their eyes if past events were physical objects that could be seen” (de la Fuente et al., 2014, p. 1684). Given that Moroccans focus more on past times and the old generation (past‐oriented), and place more value on tradition than Spaniards (future‐oriented), they are more likely to put the past in front than Spaniards.

According to the *temporal‐focus hypothesis*, people conceptualize either the future or the past as in front of them to the extent that their culture (or subculture) is future oriented or past oriented. Thus, space–time mappings in people's minds are conditioned by their cultural attitudes toward time, which are dependent on attentional focus and can be independent of the way space–time mappings are lexically expressed in language (de la Fuente et al., 2014).

Inspired by the *temporal‐focus hypothesis*, when seeking evidence for how Chinese cultural values toward time can influence Chinese people's sagittal space–time mappings, there are also surprisingly contradictory findings regarding the temporal focus of Chinese culture. Some studies have suggested that Chinese people show a tendency to be future‐oriented (e.g., Brislin & Kim, 2003), but others argue that Chinese are primarily past‐oriented (e.g., Ji, Guo, Zhang, & Messervey, 2009). Evidence for the latter would be that Chinese perceive objects in the past as being much more valuable than Americans do (Guo, Ji, Spina, & Zhang, 2012). Nevertheless, how exactly a Chinese cultural temporal‐focus of attention influences their space‐time mapping is still unclear. In short, studies on Mandarin speakers' mental sagittal space–time mappings seem to be inconclusive.

Given the fact that Mandarin speakers use lexically expressed sagittal space–time metaphors that may influence speakers' temporal orientation or perspective‐taking (e.g., Gu et al., 2019; Lai & Boroditsky, 2013; Xiao et al., 2018), and given that cultural attitude toward time has been shown to be an important determiner of individuals' space–time mappings (which can be independent of linguistic space–time metaphor; see, for example, Moroccans, de la Fuente et al., 2014), the study of Mandarin creates an opportunity to test how language‐based space–time mappings interact with more culturally motivated temporal‐focus‐based mappings. Such an attempt can not only provide a better understanding on Chinese people's metaphorical sagittal temporal orientation, but may also enrich our understanding of how language and culture can co‐influence people's mental space–time mappings.

To achieve this general research aim, in the current study, we will study how Chinese people conceptualize time on the front‐back axis, that is, whether they conceptualize the past as in front of or behind them. In particular, we will conduct a survey on Chinese cultural values toward time (temporal‐focus of attention), and several experiments to investigate Chinese people's spontaneous temporal gestures and action performances in space–time mappings, while taking different Mandarin sagittal spatial metaphors for time into consideration.

First, in Experiment 1, we will look at spontaneous gestures, as these can be seen as a “vivid and naturalistic source of evidence for the use of space in abstract reasoning” (e.g., Casasanto & Jasmin, 2012; Cienki, 1998; Cooperrider, Gentner, & Goldin‐Meadow, 2016; Cooperrider & Núñez, 2009; Núñez & Sweetser, 2006), and can provide a window into spatial cognition (Alibali, 2005; Goldin‐Meadow, 2003; Gu, Mol, Hoetjes, & Swerts, 2017; Hostetter & Alibali, 2008; Kita, Danziger, & Stolz, 2001; Walker & Cooperrider, 2016). A previous case study described how a Mandarin speaker employs the sagittal axis to gesture about time (Chui, 2011), but that was an observation from one participant only. The present study will do a quantitative research on a larger, more representative sample of Chinese speakers' sagittal temporal gestures.

Second, to corroborate the patterns of space–time mappings observed from spontaneous gestures, we will adopt a temporal performance task in Experiment 2, which has been used in several previous studies to explicitly test people's mental space–time mappings (e.g., de la Fuente et al., 2014; Li & Cao, 2018a, 2018b). Particularly, we are interested in whether these mappings are affected by different space–time metaphors. Third, with a survey on Chinese people's temporal focus of attention, we will investigate the influence of language on spatial‐temporal mappings while controlling for Chinese attitude toward time (Experiment 3).

Finally, we will perform a cross‐cultural comparison of Chinese, Moroccans, and Spaniards regarding their cultural temporal‐focus of attention (comparing data we collected ourselves with data obtained in previous studies), and we will explore whether variation in temporal‐focus may influence corresponding space–time mappings.

## 2 Experiment 1: Do Chinese people spontaneously gesture the past to the front?

As spontaneous gestures have been argued to provide a window into people's mental space–time mappings (e.g., Casasanto & Jasmin, 2012), in Experiment 1, we used a word definition task that has previously been used by Gu, Mol, et al. (2017) and Gu et al. (2019) to elicit Chinese people's speech accompanying gestures about time. This task also enabled us to study the possible effect of temporal language on co‐speech temporal gestures. The goal of this experiment is twofold: (a) we will investigate whether Mandarin speakers systematically produce sagittal gestures and examine the temporal orientation of the sagittal temporal gestures (e.g., whether the past is gestured to the front or back); and (b) we will try to further explore the relationship between the temporal orientation of sagittal gestures and the accompanying temporal language.

### 2.1 Method

#### 2.1.1 Participants

A total of 34 monolingual Mandarin speakers (*M*_age_ = 33.79 years, *SD* = 7.58, 12 males) participated as speakers in an experiment conducted in Rizhao, China. Three participants were excluded in later analyses as they did not produce any gestures. Participants' education level was about middle to senior high school (self‐reported, *M* = 2.55, *SD* = 0.96, 1‐primary school; 2‐middle school; 3‐high school; 4‐college; 5‐university).

#### 2.1.2 Materials and procedure

**Figure 2:**
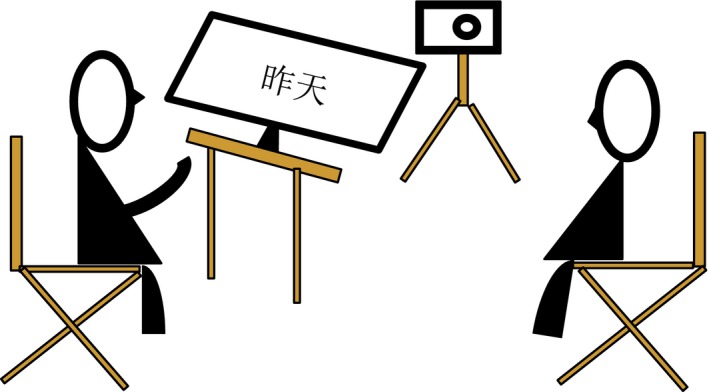
Schematic illustration of the experimental set‐up.

We constructed 12 wordlists, each containing two to four expressions that were thematically related (e.g. “yesterday,” “today,” and “tomorrow”). Five wordlists were about time conceptions, which in total consisted of 13 temporal expressions (see Appendix); the rest were fillers. The experiment was ostensibly set up as a test of speakers' short‐term memory and addressees' long‐term memory. As speakers, participants were asked to remember each wordlist shortly after they had seen them twice; they had to tell and explain the words (i.e., give definitions) as explicitly as possible to addressees who could ask them clarification questions (Fig. 2; for more details, see Gu, Mol, et al., 2017 and Gu et al., 2019). The addressees were told to “remember as many descriptions of the speaker as possible for a later memory test” (the latter test, in fact, did not take place). Gestures were not mentioned at any moment. The experiment was audio‐video recorded after participants had explicitly given their informed consent. Debriefing responses indicated that participants had not been aware that the study was about speakers' gestures.

#### 2.1.3 Coding of the data

Temporal gestures were annotated in ELAN (Lausberg & Sloetjes, 2009). A first coder performed an initial coding, viewing the entire video with the accompanying audio. The axes of gestures were coded as vertical, lateral, or sagittal, and the temporal orientation of each axis was indicated as well (Casasanto & Jasmin, 2012; Gu, Mol, et al., 2017; Gu, Zheng, et al., 2019). The time words accompanying temporal gestures were also transcribed.

The present study focused on sagittal temporal gestures; of course, people can also use the lateral and vertical axes to position time in space (e.g., Boroditsky, 2001; Fuhrman et al., 2011), also in Chinese, but these were not addressed here (see a more detailed discussion about Chinese people's lateral and vertical temporal gestures in Gu et al., 2013; Gu, Mol, et al., 2017; Gu et al., 2019). Therefore, vertical or lateral gestures were all treated as “non‐sagittal” in the later analyses of this study. Accordingly, the temporal expressions were coded as sagittal or non‐sagittal. Sagittal words included temporal words explicitly having overt sagittal spatial references to “front” (前/qián) and “back” (后/hòu) (e.g., “后年/hòu‐nián,” literally *back year*, the year after next year). All the other temporal words were coded as non‐sagittal. Thus, we obtained binary scores for the axis of each temporal gesture (sagittal or non‐sagittal), for the temporal orientation of each sagittal gesture (a future‐in‐front/past‐at‐back or past‐in‐front/future‐at‐back mapping), and for the lexically expressed references to time accompanying each temporal gesture (sagittal or non‐sagittal words).

In total, we obtained 507 temporal word‐gesture tokens that contained both a word and a gesture. The inter‐coder reliability of the annotation was established by having a naïve second person code videos of 10 randomly chosen participants (37.7% of all temporal gesture data). The two coders agreed on the gesture axes judgement on 91.1% of the tokens (*N* = 191), Cohen's Kappa = 0.84 (referring to “Excellent” agreement). In cases of disagreement, the two coders discussed and reached agreement on the labels, which were then used for the final analysis.

### 2.2 Results and discussion

**Figure 3:**
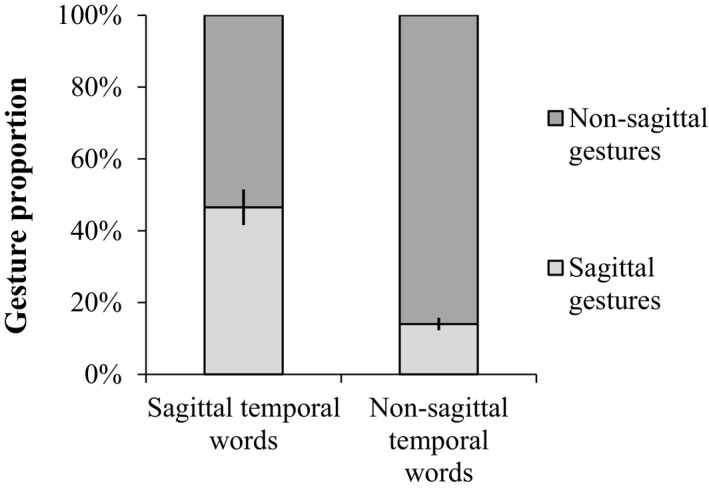
The proportion of sagittal and non‐sagittal temporal gestures (with SE error bars) when accompanying sagittal and non‐sagittal temporal words.

First, we found that Chinese people indeed spontaneously produced sagittal temporal gestures (*N* = 104), which accounted for 20.51% of all temporal gestures. Interestingly, as shown in Fig. 3, when participants were uttering sagittal temporal words, almost half of their co‐speech temporal gestures (46.53%) were produced on the sagittal axis. However, the proportion of sagittal gestures decreased to 14.04% when participants were uttering non‐sagittal temporal words.

The proportion of sagittal gestures was analyzed as a function of sagittal temporal words using a binary logistic regression for panel data, which considered multiple responses from the same participants and took individual differences, like age, education, and gender, into account. The results showed that participants were more likely to produce sagittal temporal gestures when uttering sagittal temporal words than when uttering non‐sagittal temporal words, *χ* ^2^(1) = 29.28, *N* = 507, *p* < .001, β = 1.96, 95% CI = [1.25, 2.67], while controlling for age, education, and gender. This indicated that sagittal gestures are influenced by the accompanying temporal words.

Furthermore, when focusing on the temporal orientation of sagittal gestures, the results showed that apart from gesturing the past to the back and the future to the front (past‐at‐back/future‐in‐front gestures) (50.96%), Chinese people gestured the past to the front and the future to the back (past‐in‐front/future‐at‐back gestures) (49.04%). In comparison to previous studies with English speakers, the proportion of past‐in‐front/future‐at‐back gestures by Mandarin speakers is surprisingly high. English speakers instead predominately produce sagittal temporal gestures with past‐at‐back/future‐in‐front mappings (about 80%, Casasanto & Jasmin, 2012). Based on such gestural behaviors, and based on the claim that temporal gestures can reveal people's conceptualization of time (e.g., Casasanto & Jasmin, 2012; Cienki, 1998; Núñez & Sweetser, 2006), this finding suggests that Chinese sometimes can visualize time in space as the Aymara do ( past‐in‐front/future‐at‐back).

**Figure 4:**
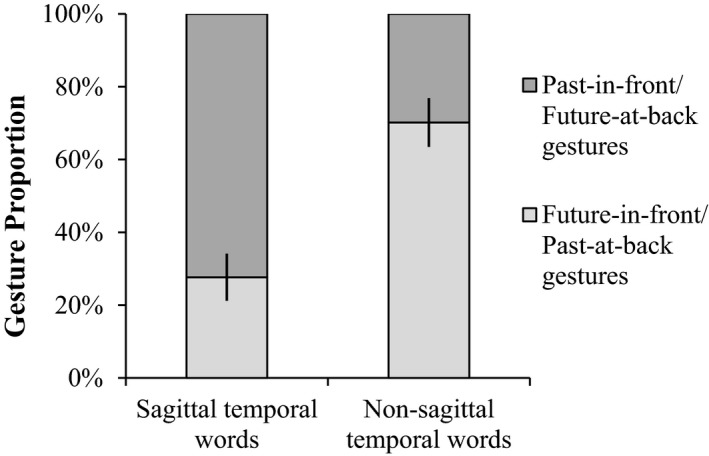
The temporal orientation of sagittal temporal gestures (with SE error bars) and the corresponding accompanying temporal words.

Interestingly, the temporal orientation of sagittal temporal gestures appears to be associated with the accompanying temporal words. In our study, 72.34% of the sagittal gestures were past‐in‐front/future‐at‐back when participants were uttering overt sagittal temporal words, whereas the proportion dropped to only 29.82% when participants were speaking non‐sagittal temporal words (Fig. 4).

This was born out by the analysis, because a regression (*N* = 104) of sagittal gesture orientation on sagittal temporal words showed that the temporal orientation of sagittal gestures was influenced by the accompanying temporal words. Specifically, at the sagittal axis, participants were more likely to perform past‐in‐front/future‐at‐back gestures when speaking sagittal temporal words than when speaking other temporal words, *χ* ^2^(1) = 6.64, *p* = .01 (two‐tailed), β = 5.57, 95% CI = [1.33, 9.80], while controlling for age, education, and gender.

This Chinese temporal gesture pattern was different from the pattern by English speakers from a previous study, in which there was no significant effect of the metaphorical spatial words on temporal gesture orientation (Casasanto & Jasmin, 2012). The result that Chinese people's past‐in‐front/future‐at‐back gestures were more often associated with sagittal temporal words may be due to the use of past‐in‐front/future‐at‐back space–time metaphors. Given that Mandarin sagittal words for spatial “front” (前/qián)” and “back” (后/hòu) are also used as temporal conceptions of “before/past” and “after/future,” the sagittal spatial metaphors for time can suggest past‐in‐front/future‐at‐back space–time mappings,^1^ and therefore can significantly influence the temporal orientation of sagittal temporal gestures.

However, as mentioned in the introduction (Example 2, Table 1), Mandarin does not exclusively use lexical cues to associate past with front, but also has the option to use words that suggest that the future is in front.

Additionally, some temporal expressions consist of words that do not contain spatial metaphors (neutral words, like “yesterday,” “later”). If it is the case that there is an effect of sagittal temporal words on the temporal orientation of sagittal gestures (as shown above), then compared to when uttering past‐in‐front/future‐at‐back metaphors, Chinese people are expected to perform fewer past‐in‐front/future‐at‐back gestures when they are uttering future‐in‐front/past‐at‐back metaphors, or neutral temporal words.

**Figure 5:**
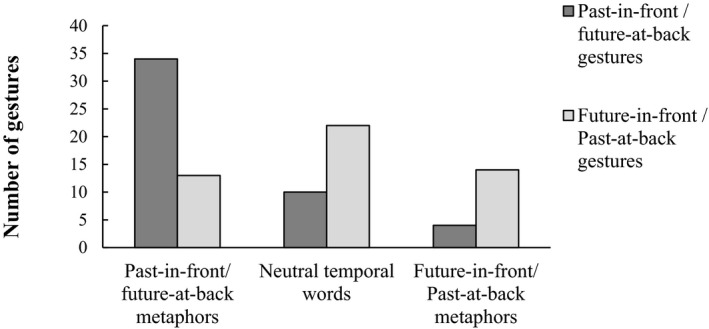
The number of past‐in‐front/future‐at‐back gestures and future‐in‐front/past‐at‐back gestures in the past‐in‐front/future‐at‐back metaphors, neutral words, and future‐in‐front/past‐at‐back metaphors conditions.

To further confirm this assumption, we sorted sagittal gestures that co‐occurred with temporal words of past‐in‐front/future‐at‐back, neutral words, and future‐in‐front/past‐at‐back metaphors (see Fig. 5) and ran a regression (*N* = 97) of sagittal gesture temporal orientation on sagittal temporal metaphors. (Note that we focused on the analysis of sagittal axis; the few cases of vertical temporal words (*N* = 7) were dropped rather than merged to any category because they were neither sagittal spatial metaphors for time nor neutral wordings. It is also inappropriate to create a new category that would cover a neglectable amount of cases only.) As predicted, the proportion of past‐in‐front/future‐at‐back gestures uttered with past‐in‐front/future‐at‐back metaphors (72.34%) was significantly higher than when uttered with neutral words (31.25%, *χ* ^2^(1) = 5.59, β*_neutral_* = −7.83, *p* = .018, 95% CI = [−14.32, −1.34]) and with future‐in‐front/past‐at‐back metaphors (22.22%, *χ* ^2^(1) = 8.20, β*_future_front_* = −12.64, *p* = .004, 95% CI = [−21.29, −3.99]), while controlling for age, gender, and education.

In short, the results of this study showed that Chinese speakers can produce sagittal gestures not only directing the past to their back but also to their front. The extent to which they performed past‐in‐front/future‐at‐back gestures was influenced by the accompanying temporal words (i.e., past‐in‐front/future‐at‐back metaphors; neutral words; future‐in‐front/past‐at‐back metaphors). Nevertheless, in Experiment 1, due to the fact that co‐speech gestures were spontaneously produced on the fly, the number of total gestures in each metaphor condition could be rather unbalanced (see Fig. 5). Therefore, in Experiment 2, we used a more controlled and explicit approach to corroborate this first set of findings.

## 3 Experiment 2: Do Chinese people place past events in front?

A temporal performance task, adapted from de la Fuente et al. (2014)'s temporal diagram task, was used to examine Chinese people's space–time mappings. This task has been shown to be an efficient and reliable paradigm to test people's sagittal mental space–time mappings in several cross‐cultural studies (e.g., Casasanto, 2009; de la Fuente et al., 2014; Li & Cao, 2018a, 2018b), and it is more explicit than our gesture study in Experiment 1 about people's mental representations.

### 3.1 Method

#### 3.1.1 Participants

A total of 114 Mandarin monolinguals (*M*_age_ = 23.64 years, *SD* = 7.98, 56 females) were assigned to three different (between‐subject) temporal word conditions (cf. Experiment 1 had three within‐subject conditions): 38 in neutral word condition, 37 in past‐in‐front metaphor/future‐at back condition, and 39 in the future‐in‐front/past‐at‐back metaphor condition. Each participant was tested individually in Rizhao, China. The mean education level of participants was between senior high school to college (*M* = 3.58, *SD* = 0.83, 1‐primary school; 2‐middle school; 3‐high school; 4‐college; 5‐university).

#### 3.1.2 Materials and procedure

**Figure 6:**
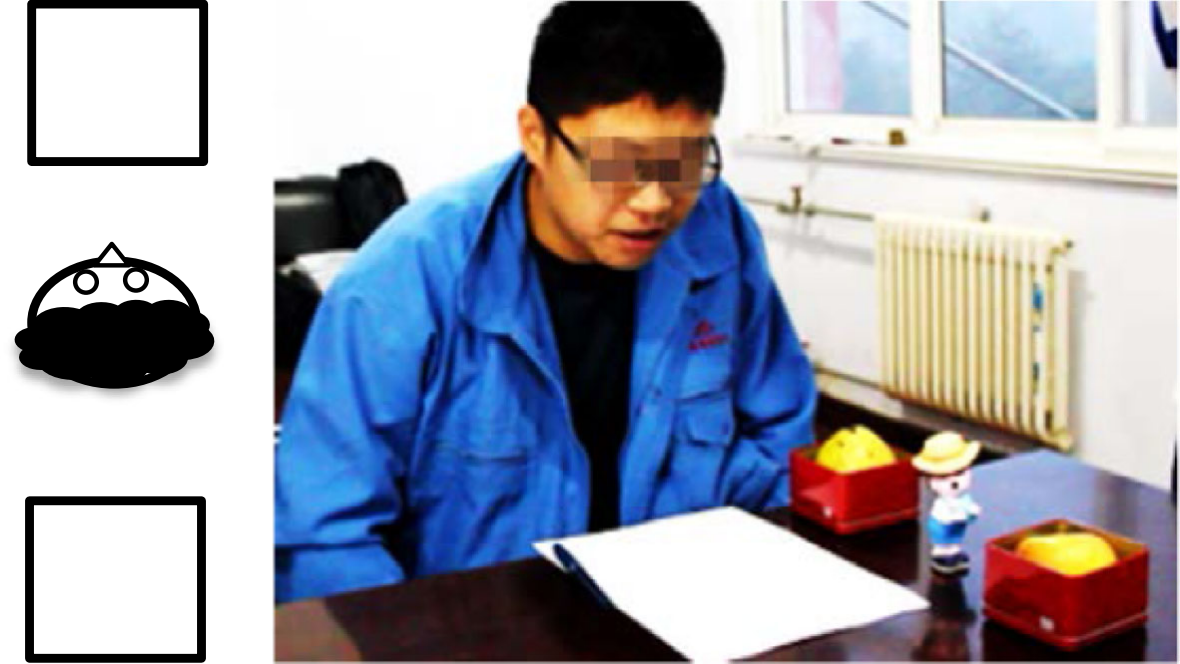
Schematic illustration of de la Fuente et al. (2014)'s temporal diagram task (left); setup of Exp. 2 (right).

Participants sat at a table on which they viewed a toy doll (named Xiaoming) positioned between two boxes. The doll and boxes were positioned on a sagittal axis from the participants' perspective, whereby the participants and the character faced the same sagittal direction (Fig. 6). The instruction presented to the participants was the same across three conditions, except, as explained below, that the wordings of temporal expressions were manipulated with the use of (a) neutral words, (b) past‐in‐front/future‐at‐back metaphors, and (c) future‐in‐front/past‐at‐back metaphors. All materials were in Mandarin.

For the neutral word condition, participants read that *yesterday* (昨天, zuó‐tiān) Xiaoming went to visit a friend who liked eating apples, and *tomorrow* (明天, míng‐tiān) he would be going to visit a friend who likes eating pears (or vice versa). Participants were given an apple and a pear and were instructed to put the *apple* in the box that corresponded to what happened at *an earlier time* and the *pear* in the box that corresponded to what would happen at *a later time*. The temporal expressions (e.g., yesterday, later) in the instructions consisted of neutral words that did not contain any lexical cues referring to space. The mentioning order of the apples and pears was counterbalanced (same for other conditions), as well as the way they were paired with temporal expressions *yesterday* and *tomorrow*.

For the past‐in‐front/future‐at‐back metaphor condition, however, the expressions about time in the instruction were changed from neutral words to explicit past‐in‐front/future‐at‐back spatial metaphors: *The day before yesterday* (前天/qián‐tiān, “front day”) Xiaoming went to visit a friend who liked eating apples, and *the day after tomorrow* (后天/hòu‐tiān, “back day”) he would be going to visit a friend who likes eating pears. Participants were instructed to put the *apple* in the box that corresponded to the *past* (以前/yǐ‐qián, *to front*, before) events and the *pear* in the box that corresponded to the *future* (今后/jīn‐hòu, *now back*, from now on) events. Note that the new pair of temporal constructs (*the day before yesterday* and *the day after tomorrow*) had a similar period of time unit as the pair of *yesterday* and *tomorrow* in the neutral word condition, both being one or two days away from the *now* moment.

For the future‐in‐front/past‐at‐back metaphor condition, the instruction was the same as that in the neutral word condition except that the neutral wording of *an earlier time* and *a later time* in the instruction were replaced with future‐in‐front/past‐at‐back metaphors. Specifically, participants were instructed to put the *apple* in the box that corresponded to *past* (过去/guò‐qù, *pass go*) events, and the *pear* in the box that corresponded to *future* (未来/wèi lái, *will/not yet come*) events.

We made two adjustments to de la Fuente et al. (2014)'s paradigm. First, de la Fuente et al. (2014) used the entities of “plant” and “animal” to represent the conceptions of “past” and “future,” whereas we used “apple” and “pear” to reduce the possible temporal thinking of an evolutionary sequence (plants came earlier than animals). Second, as Chinese people can conceptualize time vertically with “up” as “early” and “down” as “late” (e.g., Boroditsky, 2001; Gu, Mol, et al., 2017; Gu, Zheng, et al., 2019), we had participants do the task with real entities rather than letting them write them down on paper, in this way minimizing the potential projection of vertical timelines into the sagittal dimension. After this temporal performance task, we had also a brief interview asking participants why they had such placements. These interview results are addressed in the general discussion.

### 3.2 Results and discussion

In the neutral word condition, 36.8% of participants placed the fruits representing the past in front of the character and the future behind it. Even though they were still a minority, the result suggests that some Chinese indeed conceptualize the past as in front of them.

**Figure 7:**
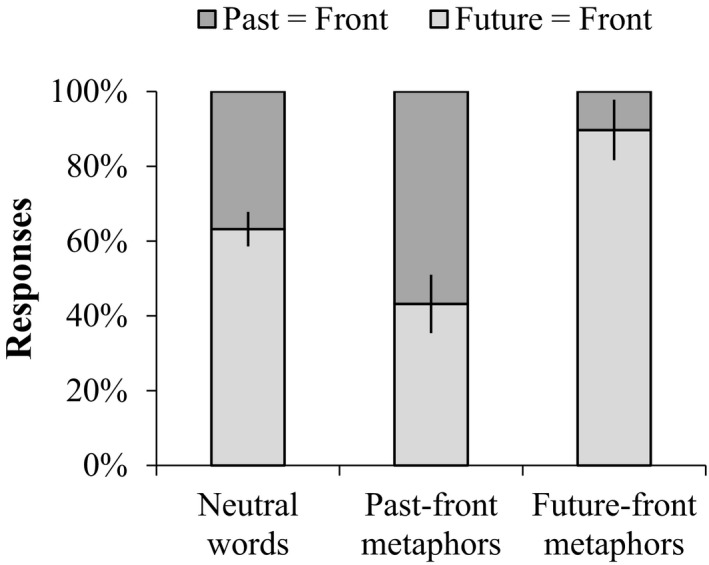
Percentage of past‐in‐front and future‐in‐front responses with SE error bars: Chinese neutral word condition, past‐in‐front/future‐at‐back metaphor condition (PFMC), and future‐in‐front/past‐at‐back metaphor condition (FFMC).

Interestingly, the participants' responses toward space–time mappings were sensitive to the different lexical conditions (Fig. 7). Specifically, in the past‐in‐front/future‐at‐back metaphor condition (PFMC), the proportion of past‐in‐front responses was 20% higher than that of in the neutral word condition (56.8% vs. 36.8%, *χ* ^2^(1) = 2.84, *N* = 75, *p* = .0917 (two tailed; but we had a directional hypothesis, so *p* = .046, one‐tailed), OR = 4.0, 95% CI = [.80, 20.04]), while controlling for age^2^ (*p* = .39) in a binary logistic regression. By contrast, in the future‐in‐front/past‐at‐back metaphor condition (FFMC), only 10.3% of participants performed past‐in‐front/future‐at‐back mappings, which was significantly lower than that of the 36.8% in the neutral condition (*χ* ^2^(1) = 6.80, *p* = .009, OR = 5.12, 95% CI = [1.50, 17.45]), and the 56.8% in the PFMC (*χ* ^2^(1) = 10.76, *p* = .001, OR = 20.30, 95% CI = [3.36, 112.46]), controlling for age (*p* = .39) in the regression (*N* = 114).

Furthermore, we recoded the temporal words of the three conditions according to the extent to which they hinted to past‐in‐front/future‐at‐back mappings (= 1 if FFMC; = 2 if neutral wording; = 3 if PFMC) and ran a regression (*N* = 114) of space–time mappings on temporal words. The results showed that temporal wording was a significant factor in predicting the probabilities that participants would perform past‐in‐front/future‐at‐back mappings (*χ* ^2^(1) = 12.20, *p* = .0005, OR = 4.64, 95% CI = [1.96, 10.96]), controlling for age (*p* = .226). It indicated that the more temporal expressions conveyed past‐in‐front/future‐at‐back mappings, the more likely Chinese would conceptualize the past in front/future at back. This again demonstrated an effect of spatial metaphors on people's mental representation of time within the Chinese culture. The results are consistent with the findings of Experiment 1 on Mandarin speakers' spontaneous time gestures.

## 4 Experiment 3: Language vs. cultural attitudes toward time

In Experiment 2, we found that linguistic space–time metaphors have a direct influence on people's mental representation of time. However, as we have reviewed in the introduction, previous research claims that space–time mappings in people's minds are also conditioned by their cultural attitudes toward time, which are dependent on attentional focus (de la Fuente et al., 2014). In Experiment 3, we investigated the roles of cultural temporal‐focus of attention and of the linguistic metaphors in shaping Chinese people's space–time mappings in a survey on Chinese people's temporal performances (a replication study of Experiment 2) and cultural attitudes toward time (temporal‐focus of attention).

### 4.1 Method

#### 4.1.1 Participants

Another 206 Mandarin speakers (*M*_age_ = 29.99, *SD* = 7.28; 61 males, 130 females and 15 gender unknown) were assigned to fulfil a 3D temporal diagram task (adapted from Experiment 2) combined with a survey of their cultural attitudes toward time (temporal‐focus of attention), with all instructions and questions written in Mandarin Chinese. Participants' education level was about university bachelor's degree (*M* = 3.11, *SD* = 0.56; 1 = Junior; 2 = High School; 3 = Bachelor; 4 = Master).

We could not ensure that participants in this experiment were monolinguals as they were recruited via social networking and their personal backgrounds were less known than participants in other experiments. Therefore, we also collected participants' English proficiency levels (*M* = 2.97, *SD* = 0.87, 5‐point‐self‐assessment), in order to be able to check whether Mandarin speakers' L2 English proficiency may influence their conceptualization of time (e.g., Lai & Boroditsky, 2013).

#### 4.1.2 Materials and procedure

First, we conducted a replication study of Experiment 2. Participants saw a 3D animated clip of a character named Xiaoming with one box behind and one box in front of him (Supplement I). Participants were randomly assigned to one of the three instruction conditions (neutral, past‐in‐front, and future‐in‐front metaphor conditions, cf. Experiment 2) and were requested to put the *apple* and *pear* in the corresponding boxes. On the next webpage, there was a test question asking participants to recall the axis (sagittal; vertical; lateral) on which the boxes were positioned with respect to the character in the clip. Those who did not indicate a sagittal axis (24 participants) were excluded from the analysis related to space–time mappings.

Next, participants were asked to fill in a temporal‐focus questionnaire to survey their cultural values toward time. Although there are alternative measurements of cultural temporal‐focus of attention (see discussions in Stolarski, Fieulaine, & van Beek, 2015; Szpunar, Spreng, & Schacter, 2014), we used the same questionnaire as de la Fuente et al.'s (2014) Experiment 4 to facilitate making direct comparisons between our results and their results with Spanish and Moroccan participants. The questionnaire was translated to Chinese and was double‐checked by a backward translation (Supplement II). It consisted of 21 statements denoting opinions about the past and the future (e.g., past‐focused: Traditions and old customs are very important for me; future‐focused: Social and cultural changes will make people happier). Participants indicated the extent to which they agreed with the statements on a 5‐point Likert scale. Those participants who did not complete the questionnaire (10) or did not provide their age (5) were excluded from the analyses that required these data. The data were collected via the survey program Qualtrics.

### 4.2 Results and discussion

First, the results of the 3D temporal diagram task showed that the proportions of past‐in‐front/future‐at‐back responses in the past‐in‐front (PFMC), neutral, and future‐in‐front metaphor (FFMC) conditions displayed a descending order (52.1%, 39.7%, and 24.6%), so in that sense replicated the results from Experiment 2 (see the three bars for Chinese in Fig. 9).

Second, according to the results from the temporal‐focus questionnaire, the Chinese participants tended to focus more on the future than on the past. On average, the past‐focused statements (*M* = 2.92, *SD* = 0.42) were rated significantly lower than the future‐focused statements (*M* = 3.16, *SD* = 0.34), *t*(195) = −5.72, *p* < .001, Cohen's *d* = 0.63, 95% CI = [−0.32, −0.16]. Following de la Fuente et al. (2014)'s proposal, for each participant, we calculated a Temporal‐focus Index [TFI = (mean of future‐focused items − mean of past‐focused items)/(mean of future‐focused items + mean of past‐focused items)], which yielded a modest future‐orientation TFI (*M* = 0.04, *SD* = 0.10) that was significantly different from 0 (*p* = .0001, Cohen's *d* = 0.4. The TFI has a scale from −1, strong past focus, to +1, strong future focus). Additionally, there were no significant differences between the TFI across three wording conditions, which also indicated that TFI was not influenced by the wording conditions.

We linked the participants' TFI to their responses toward space–time mappings (3D temporal diagram task) across three word conditions. A logistic regression (*N* = 167) of space–time mappings (dependent variable) on word conditions, keeping the TFI constant, showed that, compared to the participants in the future‐in‐front metaphor condition (FFMC), participants in the past‐in‐front metaphor condition (PFMC) (*χ* ^2^(1) = 8.30, *p* = .004, OR = 3.55, 95% CI = [1.50, 8.39]), and participants in the neutral condition (*χ* ^2^(1) = 4.30, *p* = .038, OR = 2.43, 95% CI = [1.05, 5.63]) responded significantly more with past‐in‐front/future‐at‐back mappings in the 3D temporal diagram task, while controlling for age, gender, education, English proficiency, and the instruction sequence (the order of mentioning “yesterday” first and then “tomorrow” or vice versa). However, the analysis of the influence of TFI on participants' performances in the 3D diagram task turned out not to be significant,^3^ *χ* ^2^(1) = 0.47, *p* = .49, OR = 0.29, 95% CI = [0.009, 9.67], keeping other control variables constant. Furthermore, no interaction between wording conditions and TFI was found.

The results replicated the findings in Experiment 2, showing that spatial metaphors of time have influences on Chinese people's space–time mappings, even after controlling for their cultural attitude toward time (temporal‐focus of attention). Moreover, we did not find within‐cultural evidence to support the claim that temporal‐focus of attention influences Chinese participant's space‐time mappings.

The results showed that the Chinese participants had a slightly stronger future than past orientation. Interestingly, previous studies have shown that Chinese are still more past‐oriented than Canadians or Americans (e.g., Guo et al., 2012; Ji et al., 2009). If Chinese are indeed more past‐focused than Westerners, and if the cross‐cultural differences in temporal focus predict different space–time mappings (*temporal‐focus hypothesis*, de la Fuente et al., 2014), we would expect that, at the cross‐cultural level, Chinese people are more likely to have past‐in‐front/future‐at‐back mappings than Westerners, in line with the previous findings of Moroccans. In a post hoc analysis, we compare Chinese data with the data of Moroccans and Spaniards obtained in de la Fuente et al. (2014)'s study to further scrutinize the effects of the cultural attitudes toward time on space–time mappings in a cross‐cultural context.

## 5 A post hoc analysis: A cross‐cultural comparison

### 5.1 Method

#### 5.1.1 Participants

For this analysis, we used data from 93 Moroccans (*M*_age_ = 28.57 years, *SD* = 5.72), 55 Spaniards^4^ (*M*_age_ = 20.18 years, *SD* = 2.66) (de la Fuente et al., 2014), and the 206 Chinese participants in Experiment 3. All participants had some university education.

#### 5.1.2 Procedure

All participants had followed a similar procedure to complete the temporal diagram task and the survey of cultural attitudes toward time (temporal‐focus of attention questionnaire) as described in Experiments 2 and 3.

#### 5.1.3 Comparisons and analysis

First, we used a linear regression to compare the cross‐cultural differences in temporal‐focus of attention between countries. Second, we used a logistic regression to study the cross‐cultural differences in temporal diagram placements. And third, we added TFI (Temporal‐focus Index) to the regression model to examine how cultural temporal‐focus contributes to the differences in space–time mappings.

Given that data obtained from Moroccan and Spanish groups were conducted in a neutral wording condition which could be mainly explained by their cultural temporal focus, whereas our Chinese data had three wording conditions which may introduce an extra factor of linguistic influences on space–time mappings, we limited the second and third comparisons with Chinese data only from the neutral wording condition so that participants from all cultures had the same wording condition.

### 5.2 Results and discussion

**Figure 8:**
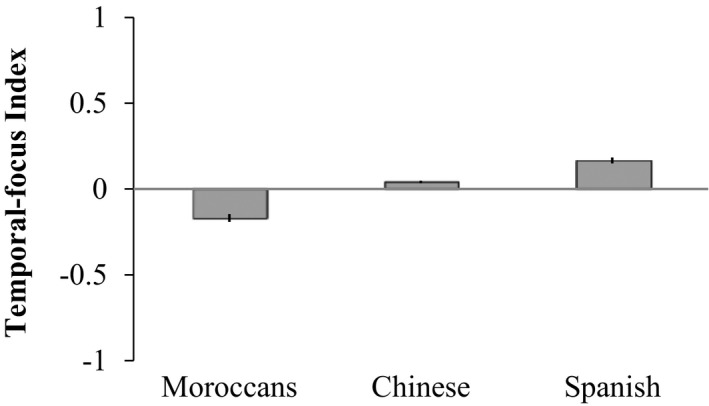
Cross‐cultural differences in normalized temporal‐focus of attention (TFI) with SE error bars. The TFI has a scale from −1 (strong past focus) to +1 (strong future focus).

First, according to the results of the temporal‐focus questionnaire, we found that there were indeed cross‐cultural differences in attitudes toward time (see Fig. 8). Using a regression (*N* = 339) of TFI on country, controlling for age, we found that, in comparisons to Chinese (*M* = 0.04, *SD* = 0.10), Moroccans (*M* = −0.17, *SD* = 0.22) had a significantly lower TFI (*F*(1, 335) = 142.77, *p* < .001, β = −0.22), and Spaniards (*M* = 0.17, *SD* = 0.13) had a significantly higher TFI (*F*(1, 335) = 8.68, *p* = .003, β = 0.07). Interestingly, the factor age, even when limiting to these younger students, was still significantly negative (*F*(1, 335) = 17.62, *p* < .001, β = −0.005), which was consistent with past research (Bylund et al., 2019; de la Fuente et al., 2014) that older participants were less future‐oriented than younger participants. In short, the results suggest that Chinese are more past‐focused than Spaniards but less past‐focused than Moroccans in their cultural attitude toward time.

**Figure 9:**
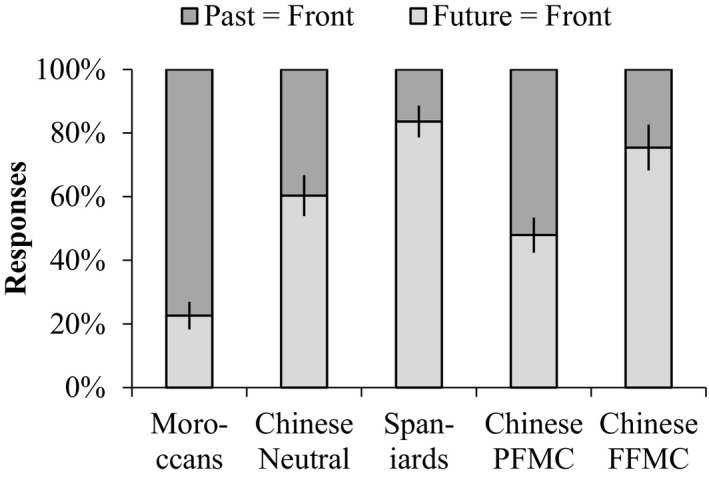
Percentage of past‐in‐front and future‐in‐front responses with SE error bars: Separately for Moroccans, Spaniards (de la Fuente et al., 2014, Exp 4), Chinese neutral word condition, past‐in‐front metaphor condition (PFMC), and future‐in‐front metaphor condition (FFMC).

Second, as for the performances in the temporal diagram task across three country groups (neutral wording condition, see the three leftmost bars in Fig. 9), a logistic regression of space–time mappings on country group showed that there were also cross‐cultural differences in responses toward space–time mappings (*χ* ^2^(2) = 59.28, *p* < .001, *N* = 206). Specifically, in comparison to the Chinese in the neutral group (39.7%), Moroccans (77.4%) were more likely to place the past in front, *χ* ^2^(1) = 20.43, *p* < .001, OR = 5.22, 95% CI = [2.55, 10.68], whereas Spaniards were less likely to do so (16.4%), *χ* ^2^(1) = 7.16, *p* = .0074, OR = 0.30, 95% CI = [.12, .72].

Furthermore, we added TFI to the regression model to study how cultural temporal‐focus contributes to the differences in space–time mappings. If temporal focus plays a role at the cross‐cultural level, the TFI is expected to be significant. If the country differences in space–time mappings are mostly captured by the cultural temporal focus, the country dummies may not be significant any more after controlling for TFI. Indeed, the TFI was highly significant (*χ* ^2^(1) = 45.88, *p* < .0001, OR = 0.000002, 95% CI = [4.73e−08, 0.00009]). However, the country group differences were *not* significant any more after controlling for the TFI.^5^ Specifically, Chinese in the neutral group no longer performed significantly different from Moroccans (*χ* ^2^(1) = 0.10, *p* = .75, OR = 1.23, 95% CI = [0.34, 4.53]) or Spaniards (*χ* ^2^(1) = 0.04, *p* = .85, OR = 0.90, 95% CI = [0.29, 2.78]).The significant TFI and these insignificant group differences indicated that the cross‐cultural differences in space–time mappings were mostly affected by the cultural‐specific temporal focus.

## 6 General discussion

In this study, we aimed to find out how linguistic expressions and cultural values toward time influence Mandarin speakers' sagittal space–time mappings by leveraging interesting properties of sagittal space–time metaphors in Mandarin Chinese. We first studied spontaneous co‐speech gestures to investigate Chinese people's implicit sagittal space–time mappings. It was found that, in addition to future‐in‐front/past‐at‐back gestures, some Chinese people produced gestures to associate the past to the front and the future to the back of them, especially in cases where they also used spatial words that suggest the past in front and the future at back (Experiment 1).

Then we used a temporal performance task (Experiment 2) to more explicitly test Chinese people's mental space–time mappings. The results confirmed that some Chinese people conceptualize the past in front and the future as behind them, and such mappings are affected by the different words of Mandarin space–time metaphors, in that sense being consistent with the results of patterns in spontaneous gestures in Experiment 1.

Furthermore, we conducted a survey on Chinese people's cultural attitudes toward time (temporal‐focus of attention) together with a 3D temporal diagram task to investigate the respective roles of the linguistic metaphors and of the Chinese cultural temporal‐focus of attention in shaping Chinese people's space–time mappings. The survey showed that our Chinese participants tended to focus a little bit more on the future than on the past. In addition, we replicated the findings of Experiment 2 that some Chinese people conceptualize the past in front and the future as behind them, and the extent to which they have such mappings is affected by the different words of Mandarin space–time metaphors, even though factors such as temporal‐focus of attention and age have been controlled for (Experiment 3). However, within the Chinese participants, we did not find evidence that the individual TFI has an influence on the sagittal space–time mappings.

Finally, with a cross‐cultural comparison in space–time mappings between Chinese, Moroccans, and Spaniards, we found that Chinese more often had past‐in‐front/future‐at‐back mappings than Spaniards and less often than Moroccans, a result which is strongly in line with predictions based on the cultural temporal‐focus of attention. In sum, we found that Chinese people's space–time mappings appeared to be the combined effect of lexical cues to space–time mappings and a culture‐specific temporal attitude toward time. The findings have several important theoretical implications to be discussed below.

### 6.1 Implications for sagittal metaphorical temporal orientation in Chinese people

Our study reveals how Chinese people conceptualize time on the front‐back axis. While some Chinese conceptualize the future as being ahead of them and the past behind them, others show the opposite pattern, and view the past as something ahead of them. More specifically, Chinese people reveal gestures and actions that reflect past‐in‐front/future‐at‐back mappings. Such mappings are peculiar, as pointing gestures referring the past to the front and the future to the back have so far only been reported in Aymara (Núñez & Sweetser, 2006).

One may argue that the Chinese past‐in‐front/future‐at‐back mappings are merely due to the lexical effect of Mandarin front/back sagittal words. That is, the explicit spatial language could simply be priming participants to make spatial responses in a way that matches the spatial expression, and thus without any space‐time mapping conceptions being involved.

However, this is unlikely for the following reasons: First, the conceptions of “前天/qián‐tiān” (“front day,” the day before yesterday) and “后天/hòu‐tiān” (“back day,” the day after tomorrow) are unambiguous in Mandarin Chinese, as they can only be interpreted as time concepts. Second, from a previous study, we know that spatial priming may not activate congruent temporal gestures. For example, “prompting [English] participants with deictic space–time metaphors did not guarantee that they would spatialize time sagittally in their gestures—not even when the prompts strongly implied a particular direction on the sagittal axis” (Casasanto & Jasmin, 2012, p. 652). There is also evidence showing that this kind of priming does not work for Chinese people. Gu et al. (2019) found that Mandarin speakers who have knowledge of Chinese Sign language will predominately produce *past‐at‐back/future‐in‐front* spontaneous gestures even when they are verbally speaking overt *past‐in‐front/future‐at‐back* space–time metaphors in their native language Mandarin Chinese. Third, even when reading an instruction in which temporal expressions consisted of *future‐in‐front/past‐at‐back* metaphors, still about one‐fifth (weighted average 19%, Experiment 2 [10.3%] and Experiment 3 [24.6%] combined) of the Chinese participants positioned the *past in front*.^6^ Furthermore, according to post hoc interviews with 37 participants^7^ who performed past‐in‐front/future‐at‐back mappings across three conditions in Experiment 2, the majority of them (29/37) explained that they had such placements because they indeed believed that the past should be in front, four of whom, similar to Aymara, argued that it was because that past is known and seen and the future yet unknown and unseen (cf. Núñez & Sweetser, 2006). The other eight participants considered that earlier events in time are in front of later events.

Thus, the results of this study contribute to the theory on Chinese people's mental orientation of time on the sagittal axis. According to Xiao et al.'s (2018) proposal, time in Chinese minds can be perceived as moving from the future to the past, where the ego faces the future (see Fig. 1). Both “early” events and “future” events are in “front,” depending on whether the perspective is taken from a time‐reference‐point or an ego‐reference‐point. From the time‐reference‐point perspective, earlier events are in front of later events, and thus the past is in the front of the timeline; from the ego‐reference‐point perspective (stationary or moving), earlier events are behind the ego, and as such the past is at the back of the ego. Therefore, “early” is in front of “late” but is still *behind* the ego.

**Figure 10:**
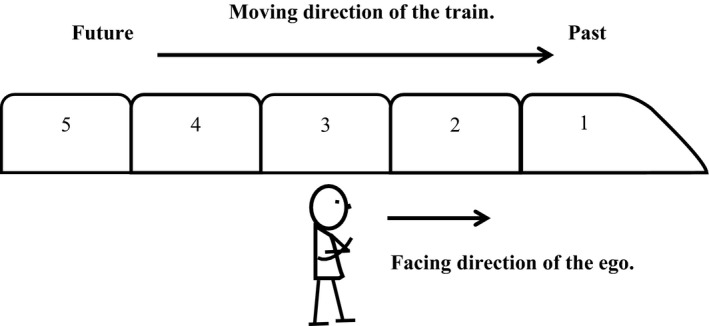
Time moves from the future to the past, but the ego stands stationary and faces the *past* (carriage 3 is now): From the time‐reference‐point perspective, earlier events (e.g., carriages 1 and 2) are in front of later events (e.g., carriages 4 and 5), and thus the past is in the front of the timeline; however, different from Fig. 1, from the ego‐reference‐point perspective, earlier events are *in front* of the ego as well, and the past is *in front of* the ego.

However, some of the empirical results in the present study may not fit into the temporal reference frames proposed by Xiao et al. (2018). For instance, some Mandarin speakers gestured the past events in front of the ego and future events behind the ego (Experiment 1), and also as mentioned above, in the post hoc interviews of Experiment 2, some explicitly explained that they felt the past to be in front of them, and the future behind them. All these results may suggest that the ego of some Chinese people can be facing the past (Ahrens & Huang, 2002; Alverson, 1994). Therefore, the sagittal timeline can not only run from the front to back (Fig. 1), but also from back to front (Fig. 10). If time is analogous to a moving train with a number of carriages, it is possible that a Chinese observer is standing still and facing the past, and the moving train is passing from the back to the front of the observer. Taking the moment when the train passes the observer as the time reference point of “now,” the carriages that have passed the observer (e.g., carriages 1 and 2) become the past, and the carriages that have not passed the person (e.g., carriages 4 and 5) are future events. There is also some linguistic evidence in Mandarin Chinese for this conjecture, such as in (1) and (2).

**Table:** 

(1)	直视自己的过去 (zhí‐shì zì‐jǐ de guò‐qù)
	straight forward look my past go
	gaze forward toward my past

**Table:** 

(2)	准备自己的后事 (zhŭn‐bèi zì‐jǐ de hòu‐shì)
	prepare for own back event
	prepare for one's own future funeral

In the above proposal, the ego is static while time is moving (Fig. 10). It shows that early/past events can be in front of an observer even when an ego is involved in the time sequence. However, despite the fact that some Mandarin speakers believe that “early” is *in front of* the ego, and “late” is *at back of* the ego, at the same time they may still conceptualize the “future” as *ahead* and the “past” as *behind* themselves. This seemingly contradictory observation regarding mental timelines cannot fully be explained by the assumptions shown in Fig. 1 or Fig. 10.

**Figure 11:**
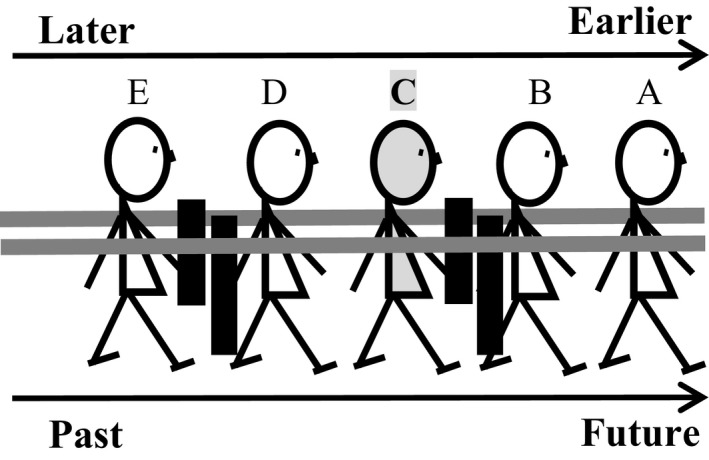
In Yu's (1998, 2012) proposal, the ego faces the future: From the ego‐reference‐point perspective, earlier people (. . . A, B) are *in front of* the ego (C), later people (D, E . . .) are *behind* the ego. However, the destination/future is also *in front of* the ego, and the past is *behind* the ego.

For those Chinese people who have a mixed mental timeline, it is likely that there is both an internal human sequence timeline and an external ego‐moving timeline (Ng et al., 2017; Xiao et al., 2018; Yu, 1998, 2012). Yu (1998, 2012) first proposes that the spatial conceptualization of temporal order of humans is similar to a human's queueing experience. In other words, it is consistent with the human's psychological reality of sequential time (Gauthier & van Wassenhove, 2016; Gentner et al., 2002; Moore, 2011). For instance, in a queue, people who are in or near the first position will be served earlier than those who are behind them (Núñez et al., 2006; Walker, Bergen, & Núñez, 2014, 2017). Specifically, suppose I am lining up in a queue (e.g., C), then within the line, the earlier people (e.g., A and B) are in front of me, and later people (e.g., D and E) are behind me (Fig. 11). However, for myself, as well as for all other people in the queue, the “future” is in front of me, as the path leading to the destination ahead, whereas the “past” is behind me, as the path I have taken to arrive at my current position (“now”). As such, both “earlier” and “future” can be in front of the ego, and both “late” and “past” can be behind the ego. Thus, the present study provides the first experimental evidence for this proposal that combines perspectives.

### 6.2 Implications for theoretical accounts of space–time mappings

This study extends our knowledge on cross‐cultural differences in space–time mappings. The results that Chinese more often have past‐in‐front/future‐at‐back mappings than Spaniards and less often than Moroccans can largely be explained by the differences in culture‐specific temporal‐focus of attention (TFI). This provides strong evidence supporting the *temporal‐focus hypothesis*, which proposes that space–time mappings in people's minds are conditioned by their cultural attitudes toward time (attentional focus) (de la Fuente et al., 2014).

Furthermore, de la Fuente et al. (2014) show that whether people conceptualize the past as behind and the future as ahead of them can vary independently from the way time is linguistically expressed in terms of spatial metaphors. By contrast, we show that, within the Chinese culture, the propensity of past‐in‐front/future‐at‐back mappings is sensitive to the linguistic variety of Mandarin space–time metaphors. For example, past‐in‐front/future‐at‐back sagittal temporal language (e.g., 前天/qián‐tiān, “front day,” the day before yesterday) will increase the probability of past‐in‐front/future‐at‐back space–time mappings while controlling for temporal‐focus. Therefore, our study shows that, irrespective of cultural temporal‐focus, sagittal space–time metaphors in language can indeed influence whether Chinese people map the past to the front or back.

Moreover, within the Chinese sample we did not find evidence supporting the *temporal‐focus hypothesis* (de la Fuente et al, 2014). Another recent study, Bylund et al. (2019), even failed to find evidence at a cross‐cultural level. Specifically, Afrikaners are found to be significantly more past‐focused than English speakers but the two groups do not differ in their front‐back mappings. Thus, it appears that the significant relationship between temporal‐focus and sagittal space–time mappings may not be found in some cases. There may exist other factors, which may influence the effect of TFI on space–time mappings. Indeed, some recent studies have shown that individuals' temporal focus and the corresponding space–time mappings are also affected by such factors, as whether one is pregnant, whether one's religious belief is Buddhism or Taoism, and whether the temporal diagram task is conducted at the beginning of a new year or on the Tomb Sweeping Day, etc. (Li & Cao, 2018a, 2018b, 2018c).

Put together, the space–time mappings seem to be influenced by an increasing number of complex factors such as culture, language, bodily experience, religion, and individual attention to time; the weight of each factor and the interplay between different ones open up future research.

### 6.3 Implications for theories on the relation between language and thought

The findings of this study provide some new insights into the relation between language and thought, especially regarding the influence of linguistic context on thinking (e.g., Bylund, & Athanasopoulos, 2017; Lai & Boroditsky, 2013; Slobin, 1996). Results from our study of spontaneous gestures and of the temporal performance task show that there is a direct effect of linguistic metaphors on people's reasoning about time. For example, past‐in‐front/future‐at‐back temporal language is more likely to activate past‐in‐front/future‐at‐back gestures/mappings. Such linguistic effect can be persistently significant even after controlling for factors of culture‐specific temporal‐focus of attention, age, education, and English proficiency (Experiment 3).

However, we need to note an important caveat that what is true about the influence of sagittal language in Mandarin on people's space–time mappings may not generalize to other languages. Many other language groups may not have the linguistic variety of space–time metaphors that Mandarin speakers have access to, and speakers of other language may not spatialize time consistent with linguistic metaphors the way Mandarin speakers do. For instance, in English sagittal space–time metaphors suggest that the future is ahead of the speaker and the past is behind, but English speakers often laterally gesture the past to the left and the future to the right even when speaking the overt sagittal temporal language,^8^ and do not make any systematic use of the sagittal axis at all^9^ (e.g., Casasanto & Jasmin, 2012; Walker & Cooperrider, 2016; Yap & Casasanto, 2018). Their mappings can mainly be explained by the left‐to‐right writing direction (Casasanto & Jasmin, 2012), but hardly explained by the sagittal future‐in‐front/past‐at‐back language in English.

Additionally, one may wonder why English speakers are also hardly influenced by the linguistic past‐in‐front/future‐at‐back mappings, given that in English the temporal “before” and “after” can also be used as spatial “front” and “back.” However, English “before” and “after” are commonly used as temporal expressions, whereas “purely spatial uses of ‘before’ and ‘after’ are rare” (Casasanto, 2016, p. 70). In fact, to express the spatial concepts of *front* and *back* in English, it is more prevalent to use “front/back” or “ahead/behind” than to use “before” and “after.”

By contrast, in Mandarin, “前/qián” and “后/hòu” are often used to express temporal concepts of “before/past” and “after/future,” while at the same time they are also the *only* words for the purely spatial use of “front” and “back.” Given that in Mandarin, most words expressing temporal past and future consist of past‐in‐front/future‐at‐back metaphors (Chen, 2007; Peng, 2012), given the frequent spatial use of “前/qián” and “后/hòu” (unlike the rarely spatial use of “before” and “after” in English), and given that people use space to conceptualize time, Mandarin speakers may well be more likely to establish the front‐back space–time mappings than English speakers.

A further question is whether the past‐in‐front/future‐at‐back mappings in Mandarin have an effect of habitual thinking on speakers' conceptualization of time. According to Slobin's (1987) “thinking‐for‐speaking” hypothesis, habitual speech patterns may influence thinking online, during linguistic processing. When speakers use certain speech patterns repeatedly, they may form habitual language‐specific conceptual schemas.

Given that in Mandarin most words expressing temporal past and future consist of past‐in‐front/future‐at‐back metaphors (Chen, 2007; Peng, 2012; Experiment 1), if habitual use of certain space–time metaphors can indeed influence one's time conceptions (Boroditsky, 2001; Hendricks, Bergen, & Marghetis, 2018; Hendricks & Boroditsky, 2017; Li, Casaponsa, Wu, & Thierry, 2019), one possibility is that, under the influence of past‐in‐front/future‐at‐back metaphors, some Mandarin speakers may form past‐in‐front/future‐at‐back space–time mappings in the long run. Especially, we did not find evidence for the effect of individual TFI (temporal focus index) on the temporal diagram placements within the Chinese participants in the neutral‐wording condition. It appears like the proportion of past‐in‐front placements in this condition is due to the possible habitual thinking from Mandarin past‐in‐front space–time metaphors. If there is such a habitual thinking which is irrespective of TFI, given that Spanish and Arabic do not suggest any deictic past‐in‐front mappings, we expect that the Chinese group in the neutral wording condition may still be significantly different from Spanish and Moroccan groups after controlling for the TFI.

However, the cross‐cultural post hoc analysis showed that the pattern of Chinese space–time mappings in the neutral wording condition can mainly be explained by the TFI. Notably, the country group differences were *no longer* significant after controlling for TFI, which did not indicate any significant additional influence such as the habitual past‐in‐front mappings from Mandarin language. Nevertheless, the non‐significance does not necessarily mean that there is no influence from the general saliency of Mandarin past‐in‐front metaphors. Thus, in the neutral wording conditions, it remains unclear whether (or the extent to which) these mappings are also influenced from the habitual thinking via the saliency of past‐in‐front space–time metaphors.

## 7 Conclusion

In this study, we investigated how Mandarin linguistic space–time metaphors and cultural attitudes toward time can influence Chinese people's use of the sagittal space to conceptualize time. We studied Chinese people's spontaneous temporal gestures and action performances in space–time mappings, as well as surveyed their cultural temporal values. The results of cross‐cultural (not within cultural) differences in spatial mappings of time provide strong evidence for the *temporal focus hypothesis* (de la Fuente et al., 2014), given that the extent to which people conceptualize the past as behind and the future as ahead of them depends on their cultural attitudes toward time. Moreover, the results of within‐cultural differences show that linguistic expressions that refer to sagittal temporal metaphors can indeed influence Mandarin speakers' space–time mappings regardless of factors such as individual temporal focus and age. Thus, in Chinese, there are both long‐term effects of cultural temporal‐focus of attention (at least at the cross‐cultural level) and immediate effects of the space–time metaphors used to probe people's mental representations. Such findings provide a better understanding of Chinese people's mental sagittal temporal orientation and of cross/within‐cultural differences in spatial‐temporal thinking, with additional implications for the theories on the relationship between language and thought.

## Supporting information


**Supplement I:** 3D temporal diagram.

**Supplement II:** Temporal—Focus questionnaire.

## Notes

1 By contrast, the use of “前/qián” (front) and “后/hòu” (back) to convey “future” and “past” conceptions is rather rare. For instance, according to a corpus survey, only 2.75% of temporal use of “后/hou” refers to “early/before” (Peng, 2012). In this study, all sagittal temporal words produced by participants were the past‐in‐front/future‐at‐back spatial metaphors for time.

2 We controlled for age, as it may significantly influence individuals' mental space‐time mappings; that is, older people are more likely to have past‐in‐front mappings than younger people (Bylund, Gygax, Samuel, & Athanasopoulos, 2019; de la Fuente et al., 2014), though the main results of this analysis and other following analyses remained unchanged if age was dropped as a factor.

3 One reviewer mentioned that difference scores have typically a low signal‐to‐noise ratio, which may lead to the insignificance of temporal‐focus. When we replaced the TFI with the raw past‐focused score and future‐focus score separately or jointly in the regression model, the results were unchanged. Another reviewer suggested that in the non‐neutral wording conditions the TFI effect may be ruled out by the effect of past‐in‐front or future‐in‐front metaphors when we analyzed three conditions as a whole. Therefore, we ran an additional analysis of the TFI on space‐time mappings only for the Chinese participants in the neutral wording condition, and the TFI was not significant. Despite this result, the overall pattern of TFI is consistent with that of space‐time mappings for Chinese. Specifically, Chinese TFI turned out to be only a bit future‐oriented (average TFI = 0.04, only moderately higher than the cutting point of 0). This pattern is matched with the proportion of future‐in‐front/past‐at‐back mappings in the neutral condition (60.3% vs. 50%), which is also slightly more likely to be future‐in‐front mappings than the cutting point of chance level.

4 There were two groups of Spaniards (younger group, *M*_age_ = 20 years, and older group, *M*_age_ = 74 years) in de la Fuente et al.'s (2014) study (Experiment 4). We used the data of the younger Spaniards group for cross‐cultural comparisons, as they were more comparable to those of the Moroccan (*M*_age_ = 29 years) and Chinese (*M*_age_ = 30 years) samples.

5 To eliminate the potential multicollinearity between TFI and age, age was excluded in the model; the results were unchanged if age was additionally controlled for.

6 One may refer to de la Fuente et al.'s (2014) study, where in 31% of the cases, older Spaniards mapped the past to the front. However, this is not comparable to our data of the future‐in‐front metaphor condition for two reasons. First, according to that study, such a “high” proportion of past‐in‐front mapping by old Spaniards was driven by their age (*M* = 74 years), given that older people are more likely to have past‐in‐front mappings. Second, they were obtained from the neutral wording condition which was not comparable to our future‐in‐front wording condition. Ideally, we should compare our future‐in‐front data with the data of young Spaniards who are university students of a similar age and do the task in the Spanish future‐in‐front condition. Suppose Spaniards can have this future‐in‐front wording condition, one can speculate that the proportion of past‐in‐front mappings of Spanish future‐in‐front metaphors condition is unlikely to be higher (actually it can be even lower) than that of Spanish neutral wording condition (e.g., 12% Experiment 1, de la Fuente et al., 2014), which in turn can still be lower than that of the Chinese future‐in‐front condition.

7 In total there were 39 participants who responded towards past‐in‐front mappings across three conditions in Experiment 1 (*N* = 114). The experiment was run individually. We decided to add a post‐hoc interview after we had already run several participants. Thus we had no interview with two participants who had performed past‐in‐front mappings (past‐in‐front condition), while we had the interview with all the rest who had past‐in‐front mappings in all conditions (37).

8 We also find such evidence in our Chinese participants, for example, past‐in‐front/ future‐at‐back temporal language does not always lead to past‐in‐front/future‐at‐back gestures or space‐time mappings (e.g., see Experiments 1 and 3). However, the intriguing result from the current study is that Mandarin speakers are more likely to perform past‐in‐front/future‐at‐back gestures/mappings when uttering lexical expressions that reflect past‐in‐front/ future‐at‐back sagittal concepts (e.g., 前天/qián tiān, “front day,” the day before yesterday).

9 One may argue that English speakers may also gesture according to past = front mappings, as in Casasanto and Jasmin (2012)'s study in which English speakers' sagittal spontaneous time gestures show a qualitative but statistically non‐significant preference for a past = back mapping. However, that insignificance is “because there were so few gestures on this axis, in total” (*N* = 14) (Casasanto & Jasmin, 2012, pp. 655–656). Only three sagittal gestures of the whole sample were produced with past‐in‐front/future‐at‐back mappings (Fig. 1b, p. 651). A recent replication study with a larger sample size confirms that English speakers do not spontaneously produce such sagittal temporal gestures in any systematic manner (Yap & Casasanto, 2018).

## Appendix 1 Wordlists of targeted time referents in Experiment 1

**Table:**

	Chinese	Meaning
(1)	上周, 下周 shàng zhōu, xià zhōu	Last week, next week
(2)	昨天, 今天, 明天 zuó tiān, jīn tiān, míng tiān	Yesterday, today, tomorrow
(3)	早晨, 晌午, 傍晚, 深夜 zǎo chén, shǎng wǔ, bàng wǎn, shēn yè	Morning, noon, evening, late at night
(4)	上辈子, 下辈子 shàng bèi zi, xià bèi zi	Previous life, next life
(5)	前年, 后年 qián nián, hòu nián	The year before last year, the year after next year
