# Supplementary figures and images for: Which Is in Front of Chinese People, Past or Future? The Effect of Language and Culture on Temporal Gestures and Spatial Conceptions of Time
Source: Cogn Sci. 2019 Dec 11;43(12):e12804. doi: 10.1111/cogs.12804 (PMC6916330; doi:10.1111/cogs.12804)

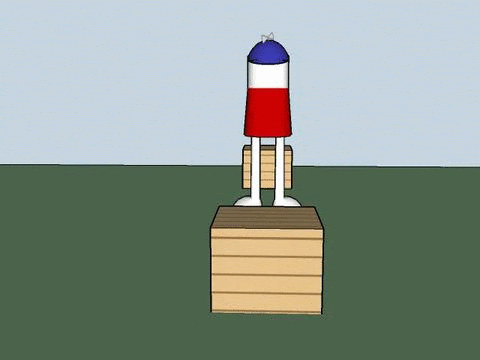

Supplement: Supplementary file 1 — Supplement I: 3D temporal diagram. [file COGS-43-na-s001.gif]
