# Supplementary material for: Which Is in Front of Chinese People, Past or Future? The Effect of Language and Culture on Temporal Gestures and Spatial Conceptions of Time
Source: Cogn Sci. 2019 Dec 11;43(12):e12804. doi: 10.1111/cogs.12804 (PMC6916330; doi:10.1111/cogs.12804)
Supplement: Supplementary file 2 — Supplement II: Temporal—Focus questionnaire. [file COGS-43-na-s002.pdf]

Supplement II: Temporal – focus questionnaire

Original English Version (de la Fuente *et al*., 2014):

1. Traditions and old customs are very important for me.
2. The young people must preserve the traditions.
3. I think that people were happier some decades ago than nowadays.
4. Present day youth need to keep the values of their parents and grandparents.
5. Older people know better than young people.
6. The right way to do things is the way in which I was taught to do them.
7. It is difficult for me to accept the cultural changes that are occurring in recent years.
8. The young people's way to have fun was better in the old times than now.
9. The traditional way of living is better than the modern way.
10. I think that the technological and economic advances in recent years are detrimental for society.
11. Respect for traditions has been lost, which is bad.
12. I understand that cultural beliefs change progressively and that we have to adapt to those changes.
13. The values and beliefs of my culture are becoming more modern, which is good.
14. I think that globalization is very positive.
15. Technological and economic advances are good for society.
16. The values and beliefs of the youth must be different from those of older people.
17. Young people do not need to learn from their elders.
18. Young people must think about the future, not in the past.
19. Traditions are not useful for the present and future society.
20. It is important to innovate and adapt to the new changes.
21. Social and cultural changes will make people happier.

Chinese Version:

1. 对我来说传统和老习俗很重要。
2. 年轻人应该保留传统。
3. 我觉得几十年前人们比现在更幸福。
4. 当今的年轻人需要保持他们父母和祖父母的价值观。
5. 长者（年长的人）比年轻人懂得更多。
6. 正确的做事方法是用我曾被教过（传授）的方法来做。
7. 这些年的文化变化让我难以接受。

8. 过去年轻人娱乐的方法比现在要好。

9. 传统的生活方式比现代的要好。

10. 我觉得近些年科技和经济的进步对社会有危害。

11. 人们对于传统的尊重已经丢失，这是很糟糕的。

12. 我知道文化信仰会逐渐发生变化，我们必须适应这些变化。

13. 人们的价值观和文化信仰正在变得更加现代化，这是很好的。

14. 我觉得全球一体化很好。

15. 科技和经济发展有利于社会。

16. 年轻人的价值观和信仰一定会和年长的人不同。

17. 年轻人不需要向他们的长辈学习。

18. 年轻人应该思考未来，而不是想着过去。

19. 传统对于当今和未来的社会没什么用。

20. 创新和适应新的变化很重要。

21. 社会和文化的变化会使人们更幸福。
